# Pre-Treatment PET Radiomics for Prediction of Disease-Free Survival in Cervical Cancer

**DOI:** 10.3390/cancers17193218

**Published:** 2025-10-02

**Authors:** Fereshteh Yousefirizi, Ghasem Hajianfar, Maziar Sabouri, Caroline Holloway, Pete Tonseth, Abraham Alexander, Tahir I. Yusufaly, Loren K. Mell, Sara Harsini, François Bénard, Habib Zaidi, Carlos Uribe, Arman Rahmim

**Affiliations:** 1 Department of Basic and Translational Research, BC Cancer Research Institute, Vancouver, BC V5Z 0B4, Canada; maziars@student.ubc.ca (M.S.); sharsini@bccrc.ca (S.H.); arman.rahmim@ubc.ca (A.R.); 2 Division of Nuclear Medicine and Molecular Imaging, Geneva University Hospital, CH-1211 Geneva, Switzerland; ghasem.hajianfar@etu.unige.ch (G.H.); habib.zaidi@hcuge.ch (H.Z.); 3 Department of Physics & Astronomy, University of British Columbia, Vancouver, BC V6T 1N4, Canada; 4 BC Cancer—Victoria, Victoria, BC V8R 6V5, Canada; cholloway@bccancer.bc.ca (C.H.); pete.tonseth@bccancer.bc.ca (P.T.); aalexander3@bccancer.bc.ca (A.A.); 5 Russell H. Morgan Department of Radiology and Radiological Sciences, School of Medicine, Johns Hopkins University, Baltimore, MD 21287, USA; tyusufa2@jhmi.edu; 6 Center for Precision Radiation Medicine, La Jolla, CA 92037, USA; lmell@ucsd.edu; 7 Department of Radiation Medicine and Applied Sciences, University of California San Diego, La Jolla, CA 92093, USA; 8 BC Cancer, Vancouver, BC V5Z 4E6, Canada; fbenard@bccrc.ca (F.B.); curibe@bccrc.ca (C.U.); 9 Department of Radiology, University of British Columbia, Vancouver, BC V5Z 1M9, Canada

**Keywords:** cervical cancer, PET/CT, radiomics, machine learning, disease-free survival

## Abstract

**Simple Summary:**

Cervical cancer continues to affect many women worldwide, with a considerable number experiencing the return of the disease after treatment. Standard imaging methods, while valuable for planning therapy, are limited in their ability to predict which patients are at higher risk of recurrence. In this study, we analyzed PET/CT scans using a computer-based approach called radiomics, which extracts detailed information about tumor shape, intensity, and texture that is not visible to the human eye. By combining these imaging features with clinical information and applying modern computer algorithms, we created models that predict the likelihood of remaining disease-free after treatment more accurately than current approaches. Our findings highlight the potential of advanced image analysis to improve treatment planning and follow-up care, moving closer to personalized strategies that may benefit women with cervical cancer.

**Abstract:**

**Background:** Cervical cancer remains a major global health concern, with high recurrence rates in advanced stages. [^18^F]FDG PET/CT provides prognostic biomarkers such as SUV, MTV, and TLG, though these are not routinely integrated into clinical protocols. Radiomics offers quantitative analysis of tumor heterogeneity, supporting risk stratification. **Purpose:** To evaluate the prognostic value of clinical and radiomic features for disease-free survival (DFS) in locoregionally advanced cervical cancer using machine learning (ML). **Methods:** Sixty-three patients (mean age 47.9 ± 14.5 years) were diagnosed between 2015 and 2020. Radiomic features were extracted from pre-treatment PET/CT (IBSI-compliant PyRadiomics). Clinical variables included age, T-stage, Dmax, lymph node involvement, SUVmax, and TMTV. Forty-two models were built by combining six feature-selection techniques (UCI, MD, MI, VH, VH.VIMP, IBMA) with seven ML algorithms (CoxPH, CB, GLMN, GLMB, RSF, ST, EV) using nested 3-fold cross-validation with bootstrap resampling. External validation was performed on 95 patients (mean age 50.6 years, FIGO IIB–IIIB) from an independent cohort with different preprocessing protocols. **Results:** Recurrence occurred in 31.7% (*n* = 20). SUVmax of lymph nodes, lymph node involvement, and TMTV were the most predictive individual features (C-index ≤ 0.77). The highest performance was achieved by UCI + EV/GLMB on combined clinical + radiomic features (C-index = 0.80, *p* < 0.05). For single feature sets, IBMA + RSF performed best for clinical (C-index = 0.72), and VH.VIMP + GLMN for radiomics (C-index = 0.71). External validation confirmed moderate generalizability (best C-index = 0.64). **Conclusions:** UCI-based feature selection with GLMB or EV yielded the best predictive accuracy, while VH.VIMP + GLMN offered superior external generalizability for radiomics-only models. These findings support the feasibility of integrating radiomics and ML for individualized DFS risk stratification in cervical cancer.

## 1. Introduction

Cervical cancer, a prominent gynecologic malignancy, ranks as the fourth most prevalent cancer among women worldwide [1]. For clinical staging, guidelines have been established by the International Federation of Obstetrics and Gynecology (FIGO) [2]. Despite advances in concurrent chemoradiotherapy, locally advanced cervical cancer (LACC) still carries a recurrence rate of approximately 35%, with a median survival of only 10–12 months following relapse [3]. The integration of 2-[^18^F]fluoro-2-deoxy-D-glucose ([^18^F]FDG) positron emission tomography with computed tomography (PET/CT) plays a pivotal role in staging, treatment planning, and overall management of cervical cancer.

Beyond staging, PET/CT has been shown in multiple studies to provide valuable prognostic information. Conventional parameters such as standardized uptake value (SUV), metabolic tumor volume (MTV), and total lesion glycolysis (TLG) have demonstrated associations with recurrence and survival outcomes. However, these metrics are not yet routinely incorporated into clinical decision-making, largely due to variability in study results and lack of standardization [4,5]. This gap underscores the need for complementary strategies, such as radiomics, which extract additional quantitative features from PET/CT images to improve risk prediction and support individualized care [6,7].

Treatment failures remain common, particularly among patients with locoregionally advanced disease, where progression rates may exceed 30% [8]. Improved tools for risk stratification are therefore needed to guide the selection of patients who may benefit from intensified treatment or novel therapeutic approaches, including immunotherapy [9]. Radiomics offers a promising solution by providing high-dimensional imaging biomarkers that capture tumor biology in greater detail than conventional PET metrics. These features, encompassing shape, intensity, and texture, reflect tumor size and geometry, metabolic activity, and intra-tumoral heterogeneity, respectively, and have been increasingly investigated as surrogate markers of prognosis and treatment response.

We therefore investigated whether pre-treatment [^18^F]FDG PET-derived metrics could enhance prognostic modeling in cervical cancer. While conventional factors such as SUV, MTV, and TLG have been proposed as prognostic indicators [10], they are not integrated into routine clinical algorithms. The FIGO/TNM staging system, particularly lymph node involvement, remains the cornerstone of prognostication [2], yet it may not capture the full biological complexity of tumors. A radiomics signature derived from pre-treatment PET scans has the potential to provide additional predictive biomarkers for recurrence, complementing established clinical parameters.

Systematically creating high-dimensional data from medical images is part of the broader field of “-omics,” which also encompasses genomics and proteomics. Radiomics specifically refers to the extraction of numerous quantitative features from medical images, including morphological (shape), intensity-based, and textural descriptors. Shape features describe tumor geometry and volumetric properties, intensity features characterize voxel intensity distributions reflecting metabolic activity, and texture features quantify spatial intensity relationships to capture intra-tumoral heterogeneity [11]. This approach has gained momentum for its potential to reveal tissue characteristics beyond visual assessment, with applications in predicting pathology, molecular subtypes, lymph node involvement, and treatment outcomes [12,13]. Prior studies indicate that texture features from [^18^F]FDG PET effectively capture heterogeneity, providing valuable prognostic insights [14].

An emerging trend combines radiomics with machine learning (ML) techniques to predict clinical outcomes [15,16,17,18,19]. In cervical cancer, radiomics from pre-treatment PET/CT has been used to predict therapy response [20,21] and survival [22,23,24]. Collectively, these features may provide stronger prognostic power than conventional PET parameters such as SUVmax, SUVmean, SUVpeak, MTV, and TLG [25]. Despite challenges arising from methodological variability and limited standardization, most studies support the predictive value of texture features [20,21]. Additionally, combining radiomics with clinical data has improved prediction of recurrence and survival [15,26], although not all studies confirm predictive value for histopathologic response [27].

In this study, we aimed to build on this body of work by developing and validating a predictive model for recurrence risk in cervical cancer using pre-treatment [^18^F]FDG PET radiomics, integrated with relevant clinical features.

## 2. Materials and Methods

**Figure 1:**
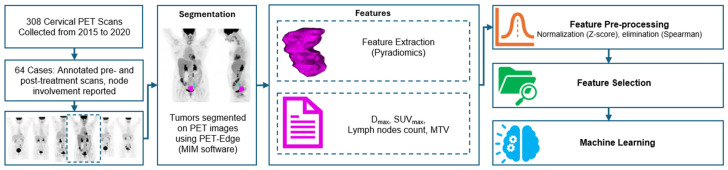
The flow chart of the presented study. A total of 308 cervical PET scans (2015–2020) were collected, of which 64 cases included annotated pre- and post-treatment scans with nodal involvement documented. Tumors were segmented on PET images using the PET-Edge tool in MIM software (Version 7.2.3). Radiomic features were extracted with PyRadiomics, complemented by clinical imaging parameters (e.g., maximum distance between primary tumor and lymph nodes and in the absence of lymph node the maximum diameter of the primary tumor: Dmax, maximum standardized uptake value: SUVmax, lymph node counts, metabolic tumor volume: MTV). Following feature extraction, pre-processing steps included normalization (Z-score) and correlation-based elimination (Spearman). Selected features were then passed through feature selection algorithms, and predictive modeling was performed using machine learning. (pink mask: tumor, blue mask: lymph node).

The flow chart of the proposed study is shown in Figure 1.

### 2.1. Patient Information

Ethics approval was granted by the Research Ethics Board of our institution. Sixty-three patients with cervical cancer, with an average age of 47.9 ± 14.5 years, that had both a pre- and post-treatment PET scan were included in this study. The radiomics analysis was applied to pre-treatment scans only, post-treatment scans were used for outcome evaluation by the clinics in this study. This cohort was gathered from patients diagnosed and treated between 2015 and 2020. In our study pre-treatment PET scans were used for prediction tasks and post-treatment PET scans were used for response evaluation by clinicians. Post-treatment scans, performed at a median of 3.53 months following radiotherapy, were clinically classified as either positive or negative for residual disease. Disease-free survival (DFS) following radiotherapy was assessed using follow-up imaging according to clinical practice guidelines [28].

The [^18^F]FDG PET/CT scans were performed as an integral component of the patient’s management plan. All imaging procedures were conducted on a Discovery 600 PET/CT scanner (GE Healthcare, Chicago, IL, USA) and reconstructed with the ordered subset expectation maximization (OSEM) algorithm including point spread function correction with the average voxel size of 3.65 × 3.65 × 3.27. There was no need for a harmonization step as only one scanner was used. Following our standardized clinical protocol, patients adhered to a 6 h fasting regimen to maintain blood glucose levels below 200 ng/dL prior to the administration of 300–400 MBq [^18^F] FDG (dependent on patient weight), with a subsequent 60 min uptake phase. The clinical and demographic data of patients are shown in Table 1. Among the eligible patients, 60 received concurrent chemoradiotherapy (chemoRT) as per standard of care. The three patients who did not complete chemoRT discontinued due to side effects (*n* = 1), liver abnormalities (*n* = 1), or renal impairment after the first cycle (*n* = 1). Additionally, 5 patients received neoadjuvant chemotherapy prior to PET imaging, but no PET scans were performed after neoadjuvant chemotherapy; therefore, these cases did not affect the radiomics analysis. The data from center 2 for the external validation is elaborated in the Supplemental File.

### 2.2. Segmentation

Tumor segmentation was performed by two experienced radiation oncologists and reviewed by a board-certified nuclear medicine physician. We used the PET-Edge gradient-based algorithm (MIM Software (Version 7.2.3)), which has been reported to yield minimal inter-observer variability and high intra-class correlation in PET tumor delineation studies. Manual refinements were applied where necessary to ensure segmentation consistency [29].

### 2.3. Radiomics Analysis

Resampling was performed using 64 bins spanning SUVs from 0 to 20 (bin width = 0.3125), a range commonly used in oncology PET imaging [30]. It is crucial to strike a balance between accurately quantifying heterogeneity and minimizing the impact of noise [31]. This range (0–20 SUV) is common in oncology with [^18^F]FDG, although adjusting the maximum value may be necessary if the uptake exceeds SUV = 20. Intensity and textural features were extracted using the Pyradiomics (version 3.0) package [32], which aligns with IBSI guidelines [11]. Initially, first-order radiomic features such as SUVmax, SUVmean, MTV, TLG, and entropy were calculated from the tumor. Furthermore, higher-order radiomic features were obtained, including those from the Neighborhood Grey Tone Difference Matrix (NGTDM) (e.g., Busyness, Coarseness, Complexity, Contrast and Strength), as well as features from the Grey Level Run Length Matrix (GLRLM) (e.g., Grey Level Nonuniformity and Run Length Nonuniformity), gray level size-zone matrix (GLSZM), gray level difference matrix (GLDM) and gray level correlation matrix (GLCM). Additionally, intensity-based area under curve of cumulative SUV-volume histogram (AUC-CSH) was integrated into the feature set. Patient age, T-stage and primary tumor features, including brachytherapy target volume, maximum distance between the primary tumor and the involved lymph nodes (Dmax), SUVmax of the primary tumor, SUVmax of LNs, number of lymph nodes, and metabolic tumor volume (MTV), were also considered. Given the consistent injected activity and acquisition protocols, along with our previous harmonization efforts [6], we determined that harmonization was unnecessary for this study. The full list of feature descriptions can be found in Table S1.

#### 2.3.1. Feature Selection

We employed six feature selection algorithms: Univariate C-Index (UCI), Minimal Depth (MD), Mutual Information (MI) [33], Variable Hunting (VH), Variable Hunting Variable Importance (VH.VIMP),all based on Random Survival Forests (RSF) [34], and Iterated Bayesian Model Averaging (IBMA) [35]. For UCI, we calculated the concordance index (C-index) of each feature using univariate Cox regression, ranked them by mean C-index over 100 bootstrap iterations, and retained the top ten. All possible combinations of these features were then evaluated, and the best-performing subset was selected.

MD, VH, and VH.VIMP rely on RSF-based ranking [34,36]. MD prioritizes features closer to the root of RSF trees, with the top seven retained. VH repeatedly partitions the data, applies RSF, and adds features iteratively until joint importance stabilizes; VH.VIMP follows the same principle but ranks by variable importance, improving efficiency. MI estimates association strength using correlation (Pearson or Spearman for continuous variables, Somers’ Dxy for survival data) [33]. IBMA iteratively applies Bayesian model averaging, retaining variables with posterior probabilities above a threshold and aggregating models weighted by posterior probability to account for uncertainty [35].

#### 2.3.2. Machine Learning Techniques

We constructed 42 survival models using combinations of six feature selection techniques and seven machine learning algorithms tailored to time-to-event prediction. ML models were selected based on their suitability for time-to-event prediction tasks, comprising Cox Proportional Hazard regression (CoxPH) [37], Cox Boost (CB) [38], Generalized Linear Model Network (GLMN) [39], Random Survival Forest (RSF) [40], GLM Boosting (GLMB) [41], and Survival Tree (ST) [42]. Ensemble Voting (EV) aggregates predictions from multiple base learners, typically including CoxPH, RSF, GLMN and GLMB in this study, by averaging their predicted risk scores. This strategy is intended to improve robustness and generalizability by combining strengths from diverse modeling approaches. To assess the efficacy of the feature selection and ML techniques, a rigorous multivariate analysis was performed, with the C-index serving as the primary evaluation metric. All modeling was performed using nested 3-fold cross-validation with 1000 bootstrap iterations per fold to ensure stability and reduce overfitting.

#### 2.3.3. Nested Cross-Validation Process

The initial step involved randomly splitting the dataset into three folds for cross-validation. In each fold, two-thirds of the data were allocated for training, while one-third was reserved for testing. Subsequently, Z-score normalization was applied to the training dataset, and the test set was normalized using the same mean and standard deviation. One of the high correlated features with a correlation coefficient > 0.95 was eliminated via Spearman’s rank, and feature selection methods were performed on the remaining features. Furthermore, to ensure that the models did not merely replicate established variables, the parameters identified in the multivariate analysis were examined for correlations with conventional measures (e.g., volume, FIGO stage, etc.). The hyperparameters for each model were fine-tuned on the training dataset using grid search and 3-fold cross-validation. Details of these hyperparameters are provided in Table S2. The best-performing model, based on the tuned hyperparameters, was then applied to the testing dataset using 1000 bootstraps for each fold. Furthermore, the statistical significance of various combinations of feature selection and ML techniques was determined through Mann–Whitney U test or Wilcoxon rank-sum test, conducted across different sets of test datasets derived from distinct outer folds.

Testing 42 FS–ML combinations was intended to systematically explore the robustness of radiomics pipelines, rather than to identify a single “winning” model. Given the relatively small cohort size, nested 3-fold cross-validation with 1000 bootstrap iterations per fold was employed to mitigate overfitting and ensure stable performance estimates.

#### 2.3.4. External Validation

The data are from the external center that were used in the previous publication [43] to predict treatment outcomes in patients with previously untreated locoregionally advanced cervical cancer. This retrospective study included 127 patients with newly diagnosed, locoregionally advanced cervical cancer who underwent pretreatment [^18^F]FDG PET/CT and received chemoradiotherapy with IMRT followed by intracavitary brachytherapy (Patient characteristics of the external dataset is summarized in Table S3). We applied the external validation on the 95 cases. The patient characteristics of the external dataset are included in the Supplementary Materials.

Patient Data: Pretreatment PET/CT scans were acquired using GE Healthcare Discovery systems. CT images were reconstructed with filtered back projection at a 512 × 512 × 1 voxel resolution. PET images were processed using one of two methods: OSEM reconstruction with 20 subsets, 2 iterations, a 4.0 mm Gaussian filter, and a 128 × 128 matrix, or OSEM with time-of-flight (TOF) and point-spread function (PSF) modeling (VUE Point FX), which applied 24 subsets, 2 iterations, a 5.0 mm Gaussian filter, a 192 × 192 matrix, and Sharp iterative reconstruction quantitation with a 9 × 6 LYSO crystal. The primary outcome was the time from diagnosis to the first occurrence of locoregional or distant recurrence or censoring, whichever came first.

Segmentation: Gross tumor volume (GTV) was manually contoured by three clinical experts based on focal hypermetabolic activity in the cervix and CT-based anatomical evidence of the primary mass. Lymph nodes were excluded from the GTV.

Feature extraction: Radiomics features were extracted using PyRadiomics (version 3.0), following the approach of Tahir et al., where features were originally extracted from five anatomical structures (primary tumor, lymph nodes, bladder, rectum, and uterus). In this study, we focused exclusively on the primary tumor, extracting shape, intensity, and texture features. A total of 193 features per structure were retained after removing redundant ones, resulting in 965 radiomic features across all structures. PET images were resampled to 5.47 × 5.47 × 3.27 mm3 using B-spline interpolation, maintaining consistency with the lowest resolution in the dataset. Feature extraction followed standard bin width settings: 0.5 SUV for PET, as recommended in prior studies. While voxel anisotropy may affect texture features, the use of standardized extraction settings ensures reproducibility and broader applicability of the findings.

We applied the trained models from the internal cohort directly to the independent dataset without any re-training or fine-tuning. Z-score normalization was applied to the external features to align feature distributions. To ensure compatibility, models relying on features not present in the external dataset, particularly SUVmax of the lymph Node, which requires lymph node segmentation—were excluded. Accordingly, validation was performed using models that did not include lymph node–specific features.

## 3. Results

We performed single-feature by Coxph analyses using key clinical variables, including FIGO/T-stage, age, Dmax, lymph node involvement, SUVmax of the primary tumor, SUVmax of the lymph node, and TMTV. The performance of each feature was evaluated independently using the concordance index (C-index), with the mean and standard deviation calculated across three folds. Among the features, SUVmax of the lymph node yielded the highest predictive performance with a C-index of 0.77 ± 0.13, followed by lymph node involvement (0.73 ± 0.12) and TMTV (0.72 ± 0.13). These findings suggest that lymph node–related features and volumetric measures may carry stronger prognostic value when used independently compared to other clinical indicators in this dataset. KM plots of single clinical features are shown in the Supplemental File, Figure S1.

Across all feature selection techniques and cross-validation folds, several features consistently emerged as top contributors. The most frequently selected feature was SUVmax node, representing the maximum SUV of the lymph node on the pre-therapy PET scan. In addition, surface area (from the morphological feature class) was frequently selected, indicating the relevance of tumor boundary complexity, and GLCM Normalized Inverse Difference Moment, a texture feature from the GLCM, which captures local intensity homogeneity. Other commonly selected features included intensity histogram Minimum Histogram Gradient, which reflects the steepest change in voxel intensity histogram, patient’s age at diagnosis, and intensity histogram Root Mean Square, a first-order statistic summarizing overall voxel intensity distribution. To assess the consistency of feature relevance, we evaluated the frequency with which each feature was selected across all folds and feature selection methods. As shown in Figure S2, features such as SUVmax node, MORPHOLOGICAL Surface Area, and GLCM Normalized Inverse Difference Moment were among the most frequently selected, suggesting strong predictive relevance and robustness across techniques.

**Figure 2:**
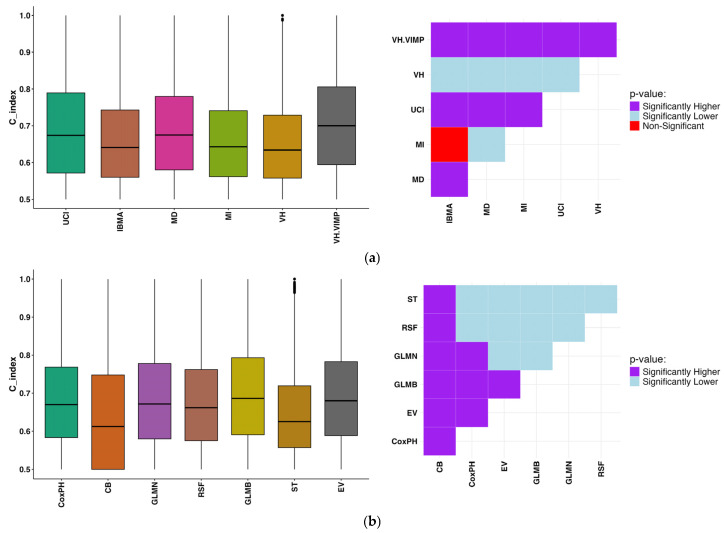
Comparison of DFS prediction performance using different features selection (**a**), and machine learning (**b**) methods in box plots (**left**) and Wilcoxon *p*-value tile plots (**right**). The c-indices are achieved through the combination of Feature Selection and Machine Learning techniques. The models in the rows were compared to those in the columns. Cyan indicates the row model had a significantly lower C-index than the column model, purple denotes the row model had a significantly higher C-index, and red shows no significant difference. (EV: ensemble voting).

The comparison of DFS prediction performance using various feature selection and machine learning techniques is illustrated in Figure 2a and Figure 2b, respectively. Among feature selection methods (Figure 2a), VH.VIMP demonstrated the highest overall performance, showing significantly superior C-index values compared to all other techniques. UCI also performed strongly and significantly outperformed MI, MD, VH, and IBMA. Although MD outperformed MI, VH and IBMA, it remained significantly inferior to UCI, and VH.VIMP. MI only surpassed IBMA, which consistently exhibited the lowest performance among all feature selection strategies.

In terms of machine learning models (Figure 2b), GLMB emerged as the best-performing method, significantly outperforming all others. Moreover, EV showed strong performance and significantly outperformed all models, except for GLMB, with which it performed comparably. RSF showed significantly higher C-index values than CB and ST. GLMN also outperformed CoxPH, CB, RSF and ST, yet was inferior to EV and GLMB. CoxPH outperformed CB, RSF and ST but fell behind the rest. CB exhibited the lowest performance across all evaluated models.

Among all evaluated combinations of feature selection and machine learning techniques, UCI_EV and UCI_GLMB, both applied to the combined feature set (clinical + radiomics), consistently demonstrated the highest mean concordance indices, with values of 0.80 ± 0.11 and 0.80 ± 0.12, respectively. These results were not only the highest in terms of predictive performance but were also found to be statistically significant (Wilcoxon rank-sum test, *p* < 0.05) when compared to other method combinations. Notably, UCI_EV appeared multiple times with exceptionally low *p*-values (e.g., *p* < 0.001), reinforcing its superior and consistent performance across multiple folds. These findings suggest that UCI feature selection, when paired with EV or GLMB, yields robust and significantly improved models, particularly when using both clinical and radiomic information (Figure 2). The C-indices for each combination of FS and ML approaches are shown in the Supplemental Materials Table S4.

**Figure 3:**
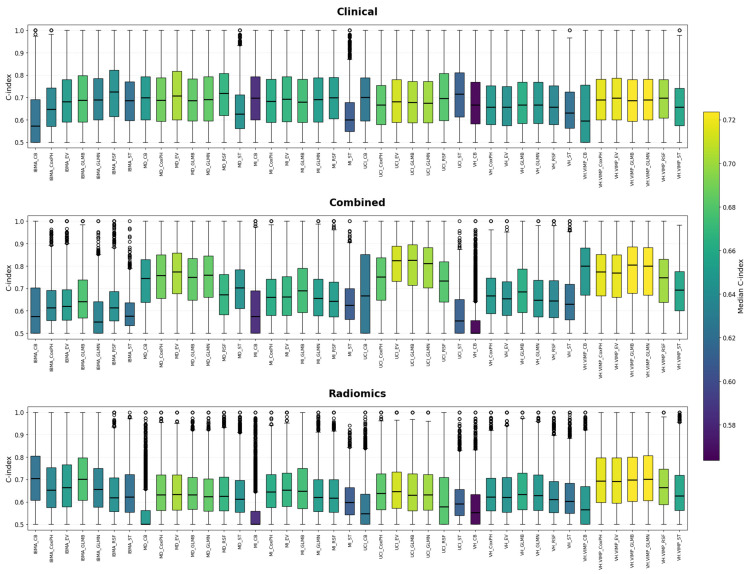
The performance evaluation of different combinations of Feature Selection (FS) and Machine Learning (ML) techniques for prediction is presented, with the assessment based on the C-index. The techniques are sorted for better visualization. The C-indices for each combination of FS and ML approaches are shown in the Supplemental Materials, Table S4.

Across the three feature categories, clinical, radiomics, and combined, distinct technique combinations consistently outperformed others in terms of both mean C-index and statistical significance (Wilcoxon rank-sum test, *p* < 0.05): Clinical features: The combination of IBMA with RSF led to the best results, with a C-index of 0.72 ± 0.12 (*p* < 0.05), showing strong discriminatory ability among clinically driven models. Radiomics features: The VH.VIMP feature selection method combined with GLMN showed the strongest performance in this group, yielding a C-index of 0.71 ± 0.13 (*p* < 0.05). Combined features: The pairing of UCI with EV and GLMB achieved the highest performance overall, with a mean C-index of 0.80 ± 0.11 and demonstrated an extremely significant difference from other methods (*p* < 0.05). These combinations of FS and ML techniques were also statistically superior to other approaches, supporting their robustness across multiple data splits and feature categories. In Figure 2 and Figure 3, the box plots illustrate the distribution of concordance indices across folds for each feature selection and machine learning combination. The adjacent Wilcoxon *p*-value tile plots show whether the performance of one model was statistically superior, inferior, or comparable to another. This combined visualization highlights both the absolute predictive accuracy and the relative statistical differences between models, supporting the conclusions drawn regarding the superiority of UCI + GLMB and UCI + EV. A concise summary of the top-performing combinations is also provided in Supplementary Table S4. Although the univariate feature SUVmax of the lymph node achieved a high C-index (0.77 ± 0.13), the Wilcoxon rank-sum test performed across 3-fold cross-validation with 1000 bootstraps per fold confirmed that the multivariate UCI + GLMB model yielded significantly higher concordance (*p* < 0.05) (see Figure 3 for more details).

**Figure 4:**
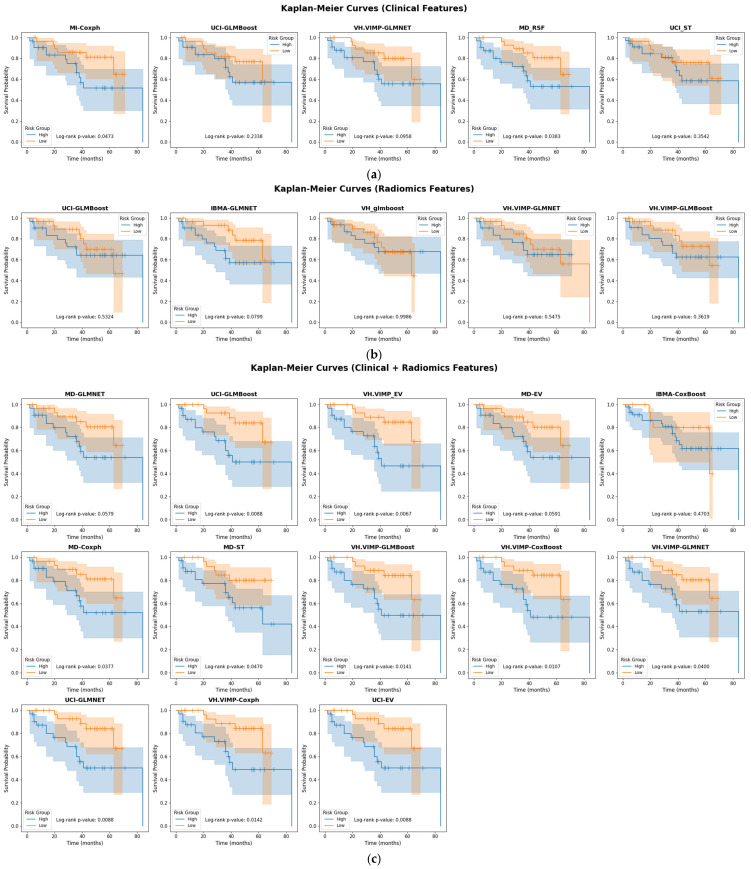
Kaplan–Meier curves corresponding to the combinations of feature selection and machine learning approaches with their corresponding log-rank *p*-values for (**a**) clinical, (**b**) radiomics and (**c**) combination of clinical and radiomics features. Kaplan–Meier curves demonstrate survival stratification between high-risk and low-risk groups for selected models. Log-rank *p*-values are reported for each comparison.

The Kaplan–Meier curves corresponding to the best models with their corresponding log-rank *p*-values are shown in Figure 4. Kaplan–Meier survival analysis demonstrated statistically significant stratification between high-risk and low-risk groups across multiple model combinations. Notably, combinations such as UCI-EV (*p* = 0.011), UCI-GLMN (*p* = 0.0067), and several models using MD-based feature selection, MD-CoxPH, MD-EV, MD-GLMN, and MD-ST, all exhibited clear survival separation with *p*-values ranging from 0.0067 to 0.04. Similarly, models built with VH.VIMP-selected features, including CB, CoxPH, EV, GLMB, and GLMN, yielded significant results (*p* < 0.05), with especially strong group separation in VH.VIMP-CB (*p* = 0.0088) and VH.VIMP-CoxPH (*p* = 0.0088).

To evaluate the generalizability of our models, we applied the best-performing feature selection and machine learning combinations—identified on the training dataset—to an independent external test dataset. The results showed that the combinations of MD with CB (MD + CB) and VH.VIMP with CB (VH.VIMP + CB) and VH.VIMP + GLMN achieved the highest C-index values (C-index = 0.64), followed closely by MI + RSF (C-index = 0.62). VH.VIMP + GLMN achieved C-index of 0.71 ± 0.13 in nested cross validation result. Notably, VH.VIMP + GLMN, which achieved a C-index of 0.71 ± 0.13 in internal nested cross-validation, showed a decline to 0.64 in external validation, reflecting reduced but still acceptable generalizability. These findings suggest that models incorporating VH.VIMP + GLMN retain acceptable generalizability when applied to unseen data and may offer applicable strategies for deployment in external clinical cohort. The Kaplan–Meier curves for external validation are shown in Figure S3.

## 4. Discussion

Our study demonstrated the prognostic significance of pre-treatment PET-based radiomics for predicting DFS in cervical cancer. Given the global burden of this disease, especially in high-risk patients or those with poor responses to standard therapy, improved prognostic tools are essential. While PET/CT is widely used in treatment planning, prognostic factors such as SUV, MTV, TLG, and radiomics features remain underutilized in clinical decision-making. Lymph node status is particularly influential in guiding treatment strategies [2] and has a substantial impact on prognosis.

Previous studies combining radiomics with clinical factors have shown improved prediction of outcomes in cervical cancer, whether using PET [23], CT [44,45], or PET/CT [46]. The prognostic utility of [^18^F]FDG PET parameters such as SUVmax, SUVmean, MTV, and TLG remains inconsistent. Although some reports suggest their value in progression-free survival [23,47] and survival outcomes in locally advanced cervical cancer (LACC) [48], others identify MTV and TLG as more reliable indicators [49], while several analyses found no independent prognostic significance [46,50]. Ferreira et al. [23] showed that neither clinical factors nor PET-derived metrics alone predicted DFS, but integration into multiparametric models substantially improved performance. Similarly, Liu et al. [46] demonstrated that combining radiomics and clinical data enhanced prognostic accuracy. Our findings are consistent with these results, showing that incorporating lymph node involvement, age, and PET-based radiomic features strengthened DFS prediction. As in prior studies [51], age emerged as an important feature, although its prognostic role remains debated [46,52].

Several features consistently emerged as strong predictors across folds and algorithms, including lymph node SUVmax, tumor surface area, and GLCM-derived homogeneity (see Figure S4). Intensity histogram features and patient age were also frequently selected, emphasizing the complementary value of clinical, morphological, and texture-based information. These features also have biological plausibility: tumor surface area reflects growth and invasion potential; GLCM homogeneity captures intra-tumoral heterogeneity, a marker of aggressive biology; and histogram-derived features summarize the intensity distribution linked to metabolic activity. While biologically relevant, radiomic features are sensitive to acquisition and reconstruction parameters. Harmonization methods and reproducibility studies, such as ComBat-based corrections, are essential for clinical translation.

These findings align with the comparative analyses shown in Figure 2 and Figure 3, where UCI-based feature selection combined with GLMB or EV consistently achieved the highest concordance indices. Wilcoxon tile plots confirmed the statistical superiority of these combinations. Overall, distinct FS–ML pairings influenced performance depending on the feature set: UCI + ST achieved the best results for clinical features (C-index = 0.72 ± 0.12, *p* < 0.001); VH.VIMP + GLMB performed best for radiomics (C-index = 0.71 ± 0.13, *p* < 0.001); and UCI + EV achieved the highest performance overall for combined data (C-index = 0.80 ± 0.11, *p* < 0.001). Among FS techniques, VH.VIMP, UCI, and VH consistently outperformed MI, MD, and IBMA, while GLMB and EV emerged as the strongest ML algorithms.

To contextualize model complexity relative to cohort size, we compared simple univariate predictors such as SUVmax of the lymph node and TMTV (C-index up to 0.77) against the best FS–ML pipelines (UCI + GLMB or UCI + EV, C-index up to 0.80). This shows that while simple metrics provide meaningful information, integrated radiomics–clinical pipelines offered incremental predictive value, supporting their evaluation despite the modest cohort size. The comparative performance of all FS–ML pipelines is summarized in Table S4, and trained models are publicly available (https://github.com/pinlab-group/CervicalSurvivalPrediction, accessed on 1 October 2025). While UCI + GLMB achieved the highest internal cross-validation performance, VH.VIMP + GLMN generalized more robustly to the external dataset. This difference likely reflects methodological variations, including the absence of lymph node features in the external dataset. Pipelines relying on node-derived predictors underperformed when such annotations were missing. By contrast, VH.VIMP tends to select features less sensitive to these variations. Thus, our results highlight the need to evaluate multiple pipelines and to tailor model choice to dataset characteristics.

We further assessed methodological quality using the METRICS checklist [53], where our study achieved a score of 77.5%, categorized as “Good.” This reflects strengths in validation and interpretability while also highlighting the need for continued work on reproducibility and harmonization. Kaplan–Meier survival analyses (Figure 4) confirmed that several model combinations, including UCI-based pipelines (UCI–EV, UCI–GLMN) and MD- or VH.VIMP-based models, significantly stratified patients into high- and low-risk groups. These findings further support the prognostic relevance of integrated radiomics–clinical approaches.

Nevertheless, limitations must be acknowledged. This was a retrospective, single-institution study with a relatively small cohort, which may limit generalizability. Although nested cross-validation and external validation were applied to mitigate overfitting, larger multi-center cohorts are needed to validate the reproducibility of radiomic signatures [54]. External validation in our study was limited by differences in acquisition protocols, segmentation (notably exclusion of lymph nodes), and feature extraction pipelines [43]. Despite harmonization using Z-score normalization, these domain shifts reduced performance compared to internal validation, reflecting the challenges of real-world variability.

At our institution, follow-up PET/CT is typically performed 3–6 months post-radiotherapy. In our dataset, 35% of recurrences were pelvic and 35% distant, highlighting both local and systemic treatment challenges. Patients with pelvic recurrence often had larger baseline tumors. These findings suggest that pre-treatment radiomics, and potentially delta-radiomics comparing pre- and post-treatment scans [6], could provide further predictive insight. Future directions should include larger prospective studies, integration of multimodal imaging such as PET/MRI [55,56,57,58], and evaluation of radiomics from both tumor and non-tumor regions [43] to refine outcome prediction.

In summary, this study demonstrates the prognostic value of PET-based radiomics combined with clinical factors in cervical cancer. Although limitations include sample size and domain shifts across datasets, our results underscore the incremental predictive power of integrated pipelines, the biological plausibility of selected features, and the potential for radiomics-driven models to guide risk stratification and treatment personalization.

## 5. Conclusions

A major challenge in radiomics is translating analytical findings into clinical decision-making for routine practice. In this study of cervical cancer patients treated with radiotherapy, we demonstrate that pre-treatment PET-based radiomic features hold prognostic value for predicting disease-free survival (DFS). Among the evaluated combinations, UCI-based feature selection combined with GLMB or EV achieved the highest predictive accuracy (C-index = 0.80) when both clinical and radiomic features were incorporated. In radiomics-only settings, VH.VIMP combined with GLMN yielded the most generalizable performance, maintaining moderate prognostic value (C-index = 0.64) in external validation despite methodological variations. External validation further confirmed the moderate generalizability of our models across different feature extraction protocols. These findings underscore the potential of radiomics and machine learning for individualized risk stratification, offering a scalable and reproducible framework to support clinical decision-making. Nonetheless, they should be interpreted as exploratory. Larger, prospective, multicenter studies with harmonized protocols are required to establish robustness, reproducibility, and clinical applicability. Future research should also examine multimodal integration (e.g., PET/MRI), delta-radiomics, and non-tumor features to improve model stability and enhance translational relevance.

## Figures and Tables

**Table 1:** Characteristics of cervical cancer patients (*n* = 64) are included in the developing (training and validation) set of this study.

Patient Characteristic	
Age (year)	47.9 ± 14.5
Node involvement in Pre-treatment PET (%)	61.9
Average nodes per patient (with node involvement)	~2
T-stage- Cervical Staging (%)	
IB1	9.5
IB2	17.5
IIA	14.3
IIB	47.6
IIIA	1.6
IIIB	9.5
Recurrence (%)	31.7
Recurrence location (%)	
Cervix/Uterus (%)	15
Pelvic (%)	35
Paraaortic nodes (%)	15
Distant (%)	35
Median follow-up (month)	3.53
Treatment	
Radiotherapy (%)	4.7 (3/63) *
Concurrent Chemoradiotherapy (chemoRT) (%)	95.2 (60/63) *
Neoadjuvant Chemotherapy before PET (%)	7.9 (5/63) *
Negative Post-treatment Scan (%)	73.0
Recurrence in patients with Negative post-treatment scan (%)	21.7

* Note: Total n = 63 used for treatment breakdown, due to data inconsistency of one patient.

## Data Availability

To safeguard our patients’ information, we are unable to disclose the data. However, for inquiries regarding the radiomics features, kindly reach out to the corresponding author.

## Supplementary Materials

The following supporting information can be downloaded at: https://www.mdpi.com/article/10.3390/cancers17193218/s1, Table S1. Feature description. Table S2. The models, R packages, and the hyperparameter settings used in this study. Table S3. Characteristics of cervical cancer patients (*n* = 95) included in external validation of this study. Table S4. Disease-Free Survival (DFS) prediction performance summarized as mean ± standard deviation (C-index) across three cross-validation folds, reported for each combination of dataset (Clinical, Radiomics, Combined), feature selection (FS) method, and machine learning (ML) model. Figure S1: Kaplan–Meier survival curves stratified by clinical features. Figure S2. The most frequently selected features in this study (not on the external center). Figure S3. Kaplan–Meier curves corresponding to the combinations of feature selection and machine learning approaches with their corresponding log-rank *p*-values on data from the radiomics features from external center. Log-rank test *p*-values are reported for each comparison. Figure S4. The correlation of the features.
