# Supplementary material for: Pre-Treatment PET Radiomics for Prediction of Disease-Free Survival in Cervical Cancer
Source: Cancers (Basel). 2025 Oct 2;17(19):3218. doi: 10.3390/cancers17193218 (PMC12523604; doi:10.3390/cancers17193218)
Supplement: Supplementary file 1 [file cancers-17-03218-s001.zip › cancers-3892254-supplementary.pdf]

SUPPLEMENTAL MATERIAL

Table S1. Feature description

Category	Feature Name	Description
Morphological (Shape) Features	**Volume / ApproximateVolume / VoxelsCounting**<br>Measures the physical volume of the lesion or the count of voxels in the ROI.<br>**SurfaceArea**<br>Calculates the total surface area of the 3D ROI.<br>**SurfaceToVolumeRatio**<br>Ratio of the surface area to the volume. It is an indicator of how "smooth" or "compact" the shape is.<br>**Compacity / Compactness1 / Compactness2 / Sphericity / Asphericity / SphericalDisproportion**<br>All are shape compactness measures. They compare the lesion's shape to an ideal sphere. For example, Sphericity close to 1 indicates a shape resembling a perfect sphere.<br>**CentreOfMassShift**<br>Assesses the displacement between the geometrical center of the lesion and the intensity-weighted center of the lesion.<br>**Maximum3DDiameter**<br>The longest straight-line distance between any two points within the lesion.<br>**IntegratedIntensity**<br>Sum of the intensity values within the tumor volume (sometimes also referred to as the product of Mean Intensity and Volume).	These features quantify the 3D geometry of the Region of Interest (ROI) and describe its size, shape complexity, and compactness that are referred to as "Shape" features in PyRadiomics (and IBSI guidelines)
First-Order Intensity-Based Features	**Mean, Median, Minimum, Maximum**<br>Basic descriptive statistics of the intensity values.<br>**Variance, StandardDeviation, InterquartileRange, Range**<br>Quantify the spread or dispersion of intensities.<br>**Skewness, Kurtosis**<br>Higher-order moments of the intensity distribution (asymmetry and tailedness).<br>**Percentiles (10th, 25th, 50th, 75th, 90th, etc.)**<br>Cut points that partition the intensity distribution.	

	**MeanAbsoluteDeviation, MedianAbsoluteDeviation, RobustMeanAbsoluteDeviation**<br>Measures of average absolute deviation around the mean or median, robust to outliers.<br>**CoefficientOfVariation, QuartileCoefficientOfDispersion**<br>Ratios that normalize dispersion to the mean or to interquartile range.<br>**Energy, RootMeanSquare, TotalLesionGlycolysis (for SUV)**<br>Derived metrics quantifying the total "power" of intensities, the square-root average of intensities, or total metabolic activity (in PET context).	
Intensity Histogram Features	**IntensityHistogramMean / Variance / Skewness / Kurtosis**<br>These parallel the first-order concepts but are specifically computed from the discrete histogram.<br>**Histogram-based Percentiles (10th, 25th, 50th, etc.), Mode, Interquartile Range**<br>Derived by analyzing the histogram shape.<br>**Entropy (Log10, Log2), Uniformity**<br>Measure randomness vs. uniform distribution of intensities in the histogram.<br>**MaximumHistogramGradient / MinimumHistogramGradient**<br>Capture how steeply the histogram rises or falls, identifying abrupt changes in intensity distribution.	These features explicitly use the histogram of voxel intensities to derive summary metrics, which may overlap with some standard first-order features but are extracted from the full histogram.
Local Intensity-Based / Local Histogram Features	**GlobalIntensityPeak(0.5 mL), GlobalIntensityPeak(1 mL), LocalIntensityPeak**<br>Aim to detect the most intense region(s) within a specified local volume (e.g., 0.5 mL or 1 mL neighborhood).<br>**IntensityPeakDiscretizedVolumeSought**<br>Similarly examines local maxima in the PET uptake distribution, often important in PET-based analysis (SUV peaks).	These features look at the local distribution of voxel intensities in addition to the global distribution.
GLCM (Gray-Level Co-Occurrence Matrix) Features	**JointMaximum, JointAverage, JointVariance, JointEntropy (Log2/Log10)**<br>Statistical measures summarizing the co-occurrence matrix.	These "second-order" texture features (often referred to as "GLCM" in

	**DifferenceAverage, DifferenceVariance, DifferenceEntropy**<br>Based on intensity differences within the GLCM.<br>**SumAverage, SumVariance, SumEntropy**<br>Based on intensity sums within the GLCM.<br>**AngularSecondMoment (ASM), Contrast, Dissimilarity, InverseDifference, InverseDifferenceMoment, Correlation**<br>Common texture metrics describing uniformity, local intensity variation, dissimilarity, etc.<br>**Autocorrelation, ClusterTendency, ClusterShade, ClusterProminence**<br>Additional measures of grouping or higher-order moments in the co-occurrence patterns.	PyRadiomics) capture heterogeneity patterns not seen by first-order statistics.
GLRLM (Gray-Level Run Length Matrix) Features	**ShortRunsEmphasis, LongRunsEmphasis**<br>Emphasize either short homogeneous "runs" of a given intensity or longer runs.<br>**LowGreyLevelRunEmphasis, HighGreyLevelRunEmphasis**<br>Emphasize runs with lower or higher intensity values.<br>**ShortRunLowGreyLevelEmphasis, ShortRunHighGreyLevelEmphasis,** etc.<br>Combine the concepts of short vs. long runs and low vs. high intensities to reflect specific texture patterns.<br>**GreyLevelNonUniformity, RunLengthNonUniformity, RunPercentage**<br>Measure how uniform runs are across different grey levels and how frequent runs are relative to total voxels.	These texture features capture the length of consecutive voxels that share the same intensity value in a given direction
GLSZM (Gray-Level Size Zone Matrix) Features	**SmallZoneEmphasis, LargeZoneEmphasis**<br>Emphasize either small or large connected zones.<br>**LowGreyLevelZoneEmphasis, HighGreyLevelZoneEmphasis**<br>Similar to GLRLM, highlight zones of lower or higher intensity levels.<br>**GreyLevelNonUniformity, ZoneSizeNonUniformity, NormalisedGreyLevelNonUniformity, NormalisedZoneSizeNonUniformity**<br>Capture how uniformly intensities and zone sizes are distributed within the ROI.	These are "GLSZM" features, capturing the size of connected regions (zones) of the same intensity value within the ROI. They complement GLRLM by considering 2D (in-slice) or 3D (volumetric) connected areas

	**ZonePercentage**<br>The ratio of the total number of zones to the total number of voxels, indicating how fragmented the lesion is.<br>**ZoneSizeEntropy, ZoneSizeVariance**<br>Describe heterogeneity or variability in zone sizes.	
NGTDM (Neighborhood Gray-Tone Difference Matrix) Features	**Coarseness, Contrast, Busyness, Complexity, Strength**<br><br>Each describes a different dimension of how intensities change when moving from one voxel to its neighboring voxels.	NGTDM features measure the difference between the intensity of a “center” voxel and the average intensity of its neighborhood. They capture more coarse or regional texture patterns.
Clinical	**tstage_cervical**<br>**age_at_dx_registry**	T stage for cervical tumors and patient’s age at diagnosis

Table S2. The models, R packages, and the hyperparameter settings used in this study. Cox Proportional Hazard regression (CoxPH), Cox Boost (CB), Generalized Linear Model Network (GLMN), Random Survival Forest (RSF), GLM Boosting (GLMB), Gradient Boosting (GB), and Survival Tree (ST).

Model	R package	Hyperparameters: range
**CoxPH**	Survival	-
**CB**	Cox Boost	maxstepno: 50-500
**RSF**	Random Forest SRC	ntree: 100, 500, 1000<br>mtry:1-10<br>node size: 1:20<br>splitrule: log-rank, logrankscore
**GLMB**	mboost	mstop: 50-500
**GLMN**	glmnet	S: 0.001-0.1<br>alpha: 0-1
**ST**	rpat	minsplit: 1-20<br>maxdepth: 1-30

Table S3. Characteristics of cervical cancer patients (n=95) included in external validation of this study: Training set of the study by Yusufaly et al. [1]. *Data for clinical features are mean, with SD in parentheses. BMI: body mass index; WBC: white blood cell count; ANC: absolute neutrophil count.

**Patient Characteristic**	
n (no. of events)	95 (23)
Stage (n)	
IA	2 (2%)
IB	23 (24%)
IIA	7 (7%)
IIB	32 (33%)
IIIA	3 (3%)
IIIB	16 (17%)
IIIC	6 (6%)
IVA	6 (6%)
IVB	0
Histology (n)	
Adenocarcinoma	23 (24%)
Squamous carcinoma	72 (76%)
Clinical features*	
Age (y)	50.6 (13.7)
BMI (kg/m²)	29.0 (6.5)
Baseline WBC (k/µL)	8.23 (2.6)
Baseline ANC (k/µL)	5.39 (2.75)
Baseline hemoglobin (g/dL)	11.6 (1.8)
Baseline platelet count (k/µL)	288 (79)

Table S4. Disease-Free Survival (DFS) prediction performance summarized as mean ± standard deviation (C-index) across three cross-validation folds, reported for each combination of dataset (Clinical, Radiomics, Combined), feature selection (FS) method, and machine learning (ML) model. Each row corresponds to a specific Dataset–FS–ML combination, and columns represent performance per fold and overall. Models are sorted by dataset for clarity. Abbreviations: UCI, Univariate C-index; VH.VIMP, Variable Hunting Variable Importance; GLMB, Generalized Linear Model Boosting; EV, Ensemble Voting; RSF, Random Survival Forest; ST, Survival Tree; CB, CoxBoost; GLMN, Generalized Linear Model Network.

DFS prediction performance using clinical features, summarized as mean ± standard deviation (C-index) across three cross-validation folds.

	Feature Set	*FS*	*Model*	*Fold1*	*Fold2*	*Fold3*	*Overall*
	Clinical	IBMA	CB	0.66±0.11	0.5±0	0.67±0.12	0.61±0.12
2	Clinical	IBMA	CoxPH	0.62±0.096	0.7±0.11	0.67±0.11	0.66±0.11
3	Clinical	IBMA	EV	0.64±0.11	0.75±0.11	0.69±0.14	0.69±0.13
4	Clinical	IBMA	GLMB	0.63±0.099	0.8±0.1	0.67±0.12	0.7±0.13
5	Clinical	IBMA	GLMN	0.65±0.11	0.76±0.11	0.68±0.11	0.7±0.12
6	Clinical	IBMA	RSF	0.63±0.097	0.78±0.11	0.76±0.12	0.72±0.13
7	Clinical	IBMA	ST	0.71±0.11	0.62±0.085	0.75±0.11	0.69±0.11
8	Clinical	MD	CB	0.65±0.11	0.78±0.091	0.67±0.12	0.7±0.12
9	Clinical	MD	CoxPH	0.65±0.11	0.78±0.1	0.66±0.12	0.7±0.12
10	Clinical	MD	EV	0.66±0.12	0.8±0.1	0.69±0.14	0.72±0.13
11	Clinical	MD	GLMB	0.65±0.11	0.77±0.1	0.66±0.12	0.7±0.12
12	Clinical	MD	GLMN	0.65±0.11	0.79±0.1	0.66±0.12	0.7±0.13
13	Clinical	MD	RSF	0.65±0.1	0.76±0.11	0.74±0.12	0.72±0.12
14	Clinical	MD	ST	0.61±0.08	0.62±0.079	0.71±0.12	0.65±0.11
15	Clinical	MI	CB	0.66±0.11	0.77±0.1	0.67±0.12	0.7±0.12
16	Clinical	MI	CoxPH	0.64±0.1	0.77±0.1	0.67±0.12	0.69±0.12
17	Clinical	MI	EV	0.65±0.11	0.77±0.1	0.68±0.14	0.7±0.13

18	Clinical	MI	GLMB	0.63±0.098	0.77±0.1	0.67±0.12	0.69±0.12
19	Clinical	MI	GLMN	0.65±0.11	0.77±0.1	0.67±0.12	0.7±0.12
20	Clinical	MI	RSF	0.71±0.12	0.66±0.1	0.75±0.14	0.71±0.13
21	Clinical	MI	ST	0.64±0.092	0.57±0.057	0.66±0.12	0.62±0.1
22	Clinical	UCI	CB	0.65±0.11	0.77±0.091	0.67±0.12	0.7±0.12
23	Clinical	UCI	CoxPH	0.64±0.11	0.72±0.1	0.66±0.12	0.68±0.11
24	Clinical	UCI	EV	0.65±0.1	0.75±0.11	0.68±0.14	0.69±0.13
25	Clinical	UCI	GLMB	0.66±0.12	0.75±0.11	0.66±0.12	0.69±0.12
26	Clinical	UCI	GLMN	0.64±0.11	0.75±0.11	0.67±0.12	0.69±0.12
27	Clinical	UCI	RSF	0.66±0.12	0.79±0.11	0.67±0.12	0.71±0.13
28	Clinical	UCI	ST	0.65±0.11	0.78±0.1	0.72±0.13	0.72±0.12
29	Clinical	VH	CB	0.62±0.097	0.78±0.1	0.65±0.091	0.68±0.12
30	Clinical	VH	CoxPH	0.62±0.099	0.76±0.11	0.64±0.09	0.67±0.12
31	Clinical	VH	EV	0.62±0.095	0.76±0.11	0.63±0.088	0.67±0.12
32	Clinical	VH	GLMB	0.62±0.099	0.78±0.1	0.65±0.091	0.68±0.12
33	Clinical	VH	GLMN	0.62±0.099	0.78±0.1	0.65±0.091	0.68±0.12
34	Clinical	VH	RSF	0.63±0.1	0.75±0.12	0.64±0.089	0.67±0.12
35	Clinical	VH	ST	0.63±0.11	0.71±0.093	0.6±0.076	0.65±0.1
36	Clinical	VH.VIMP	CB	0.63±0.1	0.77±0.092	0.5±0	0.64±0.14
37	Clinical	VH.VIMP	CoxPH	0.65±0.11	0.77±0.093	0.66±0.099	0.69±0.11
38	Clinical	VH.VIMP	EV	0.64±0.1	0.78±0.096	0.68±0.11	0.7±0.12
39	Clinical	VH.VIMP	GLMB	0.64±0.11	0.77±0.092	0.66±0.099	0.69±0.11

40	Clinical	VH.VIMP	GLMN	0.65±0.1	0.77±0.092	0.66±0.099	0.69±0.11
41	Clinical	VH.VIMP	RSF	0.66±0.1	0.75±0.099	0.69±0.12	0.7±0.11
42	Clinical	VH.VIMP	ST	0.62±0.087	0.74±0.084	0.63±0.099	0.66±0.1

DFS prediction performance using radiomics features, summarized as mean ± standard deviation (C-index) across three cross-validation folds. Each row shows the performance of a particular FS–ML combination. Fold-specific and overall scores are provided.

	Feature Set	*FS*	*Model*	*Fold1*	*Fold2*	*Fold3*	*Overall*
43	Radiomics	IBMA	CB	0.67±0.12	0.68±0.11	0.79±0.12	0.71±0.13
44	Radiomics	IBMA	CoxPH	0.63±0.1	0.71±0.11	0.68±0.12	0.67±0.12
45	Radiomics	IBMA	EV	0.63±0.099	0.7±0.11	0.71±0.14	0.68±0.12
46	Radiomics	IBMA	GLMB	0.67±0.12	0.69±0.11	0.77±0.13	0.71±0.13
47	Radiomics	IBMA	GLMN	0.67±0.11	0.67±0.11	0.68±0.12	0.67±0.12
48	Radiomics	IBMA	RSF	0.65±0.11	0.61±0.092	0.66±0.11	0.64±0.11
49	Radiomics	IBMA	ST	0.62±0.089	0.58±0.067	0.75±0.11	0.65±0.11
50	Radiomics	MD	CB	0.65±0.11	0.5	0.5	0.55±0.094
51	Radiomics	MD	CoxPH	0.68±0.11	0.63±0.091	0.64±0.1	0.65±0.1
52	Radiomics	MD	EV	0.65±0.11	0.64±0.094	0.66±0.11	0.65±0.11
53	Radiomics	MD	GLMB	0.64±0.1	0.64±0.085	0.66±0.11	0.65±0.1
54	Radiomics	MD	GLMN	0.64±0.1	0.65±0.097	0.63±0.099	0.64±0.1
55	Radiomics	MD	RSF	0.64±0.11	0.63±0.091	0.66±0.12	0.65±0.11
56	Radiomics	MD	ST	0.6±0.078	0.62±0.087	0.69±0.13	0.64±0.11
57	Radiomics	MI	CB	0.65±0.12	0.5	0.5	0.55±0.097
58	Radiomics	MI	CoxPH	0.64±0.1	0.67±0.094	0.66±0.11	0.66±0.1
59	Radiomics	MI	EV	0.64±0.1	0.67±0.095	0.67±0.12	0.66±0.11

60	Radiomics	MI	GLMB	0.65±0.11	0.61±0.085	0.74±0.12	0.67±0.12
61	Radiomics	MI	GLMN	0.61±0.086	0.62±0.084	0.68±0.11	0.64±0.1
62	Radiomics	MI	RSF	0.63±0.1	0.62±0.09	0.66±0.12	0.64±0.11
63	Radiomics	MI	ST	0.61±0.083	0.6±0.072	0.64±0.097	0.61±0.086
64	Radiomics	UCI	CB	0.64±0.11	0.61±0.088	0.5	0.58±0.099
65	Radiomics	UCI	CoxPH	0.62±0.091	0.65±0.099	0.69±0.12	0.65±0.11
66	Radiomics	UCI	EV	0.63±0.1	0.66±0.1	0.7±0.12	0.66±0.11
67	Radiomics	UCI	GLMB	0.63±0.098	0.62±0.088	0.7±0.12	0.65±0.11
68	Radiomics	UCI	GLMN	0.63±0.1	0.63±0.089	0.69±0.12	0.65±0.11
69	Radiomics	UCI	RSF	0.62±0.088	0.5±0	0.74±0.11	0.62±0.13
70	Radiomics	UCI	ST	0.61±0.088	0.59±0.072	0.63±0.1	0.61±0.089
71	Radiomics	VH	CB	0.62±0.096	0.63±0.093	0.5	0.58±0.097
72	Radiomics	VH	CoxPH	0.62±0.1	0.64±0.097	0.67±0.1	0.64±0.1
73	Radiomics	VH	EV	0.6±0.087	0.63±0.094	0.7±0.12	0.64±0.11
74	Radiomics	VH	GLMB	0.62±0.1	0.64±0.095	0.71±0.12	0.66±0.11
75	Radiomics	VH	GLMN	0.62±0.1	0.64±0.097	0.7±0.12	0.65±0.11
76	Radiomics	VH	RSF	0.62±0.098	0.61±0.082	0.67±0.12	0.63±0.1
77	Radiomics	VH	ST	0.62±0.091	0.6±0.077	0.66±0.12	0.63±0.1
78	Radiomics	VH.VIMP	CB	0.66±0.12	0.64±0.093	0.5	0.6±0.11
79	Radiomics	VH.VIMP	CoxPH	0.66±0.12	0.72±0.11	0.74±0.14	0.71±0.13
80	Radiomics	VH.VIMP	EV	0.65±0.11	0.72±0.11	0.77±0.16	0.71±0.14
81	Radiomics	VH.VIMP	GLMB	0.66±0.12	0.72±0.11	0.75±0.15	0.71±0.13

82	Radiomics	VH.VIMP	GLMN	0.66±0.12	0.73±0.12	0.75±0.15	0.71±0.13
83	Radiomics	VH.VIMP	RSF	0.65±0.11	0.69±0.092	0.68±0.12	0.67±0.11
84	Radiomics	VH.VIMP	ST	0.62±0.089	0.61±0.077	0.72±0.12	0.65±0.11

DFS prediction performance using combined clinical and radiomics features, reported as mean ± standard deviation (C-index) across three cross-validation folds. The table details each FS–ML model's performance across folds and the overall average.

	Feature Set	FS	Model	Fold1	Fold2	Fold3	Overall
85	Combined	IBMA	CB	0.68±0.12	0.5±0	0.67±0.12	0.62±0.13
86	Combined	IBMA	CoxPH	0.62±0.089	0.64±0.099	0.63±0.099	0.63±0.096
87	Combined	IBMA	EV	0.63±0.096	0.64±0.097	0.64±0.11	0.64±0.1
88	Combined	IBMA	GLMB	0.66±0.11	0.64±0.095	0.7±0.13	0.66±0.11
89	Combined	IBMA	GLMN	0.62±0.09	0.5±0	0.62±0.088	0.58±0.093
90	Combined	IBMA	RSF	0.65±0.12	0.63±0.083	0.62±0.089	0.63±0.098
91	Combined	IBMA	ST	0.64±0.076	0.55±0.041	0.59±0.069	0.59±0.074
92	Combined	MD	CB	0.65±0.1	0.78±0.1	0.79±0.11	0.74±0.12
93	Combined	MD	CoxPH	0.7±0.12	0.83±0.088	0.72±0.12	0.75±0.12
94	Combined	MD	EV	0.72±0.12	0.81±0.096	0.77±0.13	0.76±0.12
95	Combined	MD	GLMB	0.66±0.11	0.78±0.1	0.79±0.11	0.74±0.12
96	Combined	MD	GLMN	0.69±0.12	0.78±0.1	0.79±0.13	0.75±0.12
97	Combined	MD	RSF	0.7±0.12	0.7±0.11	0.64±0.11	0.68±0.11
98	Combined	MD	ST	0.63±0.094	0.76±0.082	0.71±0.11	0.7±0.11
99	Combined	MI	CB	0.65±0.11	0.67±0.1	0.5	0.61±0.11
100	Combined	MI	CoxPH	0.68±0.11	0.67±0.1	0.67±0.11	0.67±0.11
101	Combined	MI	EV	0.67±0.12	0.67±0.1	0.68±0.12	0.67±0.11

102	Combined	MI	GLMB	0.64±0.1	0.69±0.11	0.77±0.12	0.7±0.13
103	Combined	MI	GLMN	0.65±0.11	0.67±0.1	0.68±0.11	0.67±0.11
104	Combined	MI	RSF	0.64±0.1	0.67±0.1	0.66±0.12	0.66±0.11
105	Combined	MI	ST	0.64±0.095	0.64±0.089	0.64±0.096	0.64±0.094
106	Combined	UCI	CB	0.68±0.11	0.5±0	0.88±0.065	0.69±0.17
107	Combined	UCI	CoxPH	0.69±0.11	0.83±0.085	0.71±0.12	0.74±0.12
108	Combined	UCI	EV	0.71±0.11	0.84±0.087	0.87±0.085	0.8±0.11
109	Combined	UCI	GLMB	0.68±0.11	0.83±0.09	0.88±0.065	0.8±0.12
110	Combined	UCI	GLMN	0.68±0.11	0.81±0.095	0.87±0.076	0.79±0.12
111	Combined	UCI	RSF	0.72±0.11	0.77±0.1	0.7±0.13	0.73±0.12
112	Combined	UCI	ST	0.65±0.096	0.61±0.081	0.5	0.59±0.096
113	Combined	VH	CB	0.64±0.1	0.5	0.5	0.55±0.091
114	Combined	VH	CoxPH	0.72±0.1	0.65±0.096	0.65±0.093	0.67±0.1
115	Combined	VH	EV	0.67±0.099	0.66±0.1	0.66±0.1	0.66±0.1
116	Combined	VH	GLMB	0.64±0.1	0.66±0.098	0.79±0.11	0.7±0.12
117	Combined	VH	GLMN	0.64±0.1	0.7±0.11	0.64±0.092	0.66±0.11
118	Combined	VH	RSF	0.69±0.12	0.62±0.086	0.68±0.12	0.66±0.11
119	Combined	VH	ST	0.72±0.12	0.61±0.083	0.62±0.09	0.65±0.11
120	Combined	VH.VIMP	CB	0.66±0.12	0.78±0.1	0.88±0.063	0.77±0.13
121	Combined	VH.VIMP	CoxPH	0.66±0.12	0.79±0.1	0.82±0.085	0.76±0.12
122	Combined	VH.VIMP	EV	0.66±0.12	0.77±0.1	0.83±0.088	0.75±0.13
123	Combined	VH.VIMP	GLMB	0.66±0.12	0.8±0.099	0.88±0.063	0.78±0.13

124	Combined	VH.VIMP	GLMN	0.66±0.12	0.78±0.1	0.88±0.063	0.77±0.13
125	Combined	VH.VIMP	RSF	0.64±0.1	0.76±0.11	0.81±0.088	0.74±0.12
126	Combined	VH.VIMP	ST	0.61±0.077	0.77±0.085	0.69±0.099	0.69±0.11

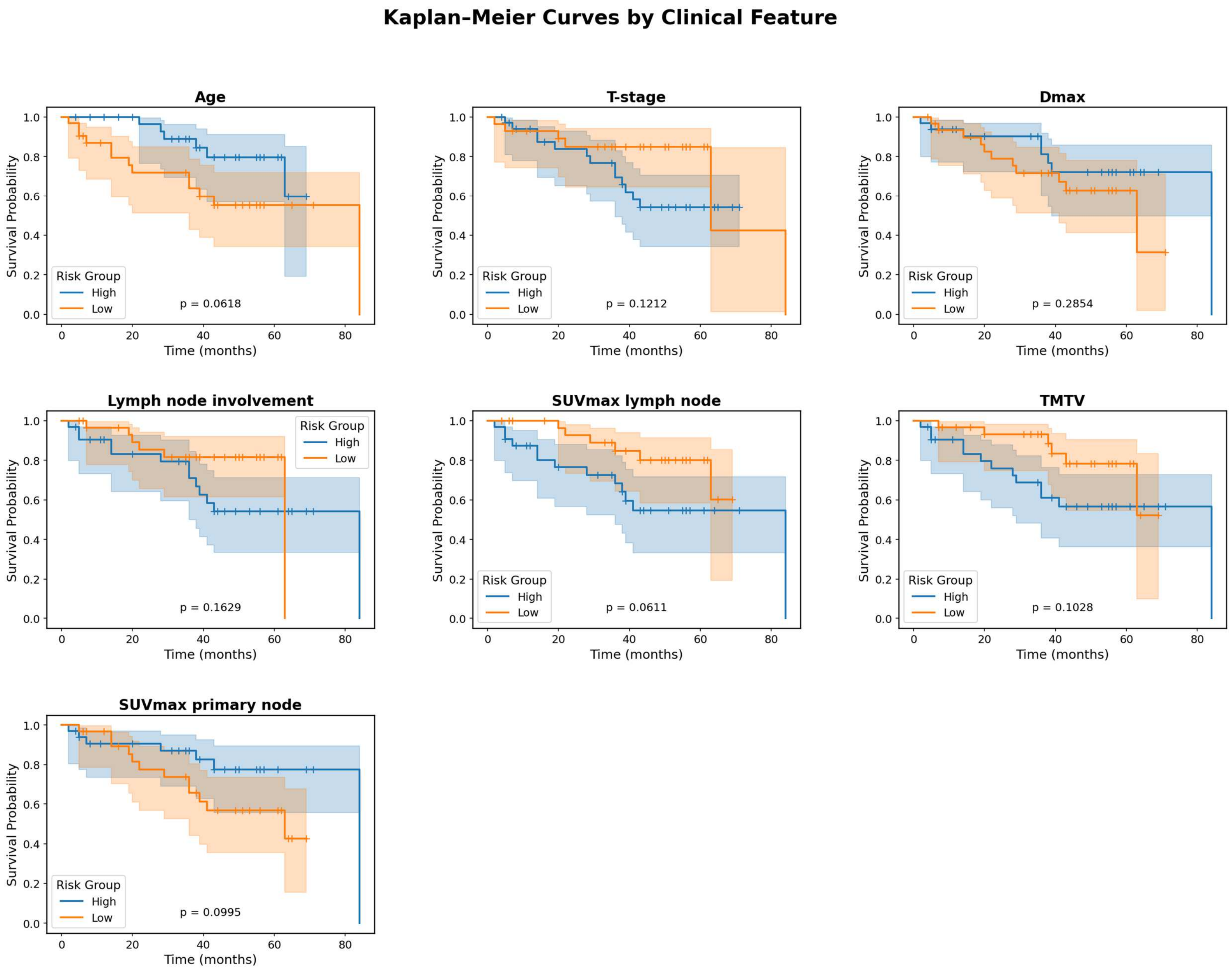
Kaplan–Meier Curves by Clinical Feature
Age
p = 0.0618
T-stage
p = 0.1212
Dmax
p = 0.2854
Lymph node involvement
p = 0.1629
SUVmax lymph node
p = 0.0611
TMTV
p = 0.1028
SUVmax primary node
p = 0.0995
Risk Group
High
Low
Survival Probability
Time (months)

Figure S1: Kaplan–Meier survival curves stratified by clinical features. Log-rank test p-values are reported for each comparison. Features shown include age, T-stage, Dmax, lymph node involvement, SUVmax of lymph nodes, and total metabolic tumor volume (TMTV). No clinical feature reached statistical significance, though age and SUVmax demonstrated borderline separation between groups.

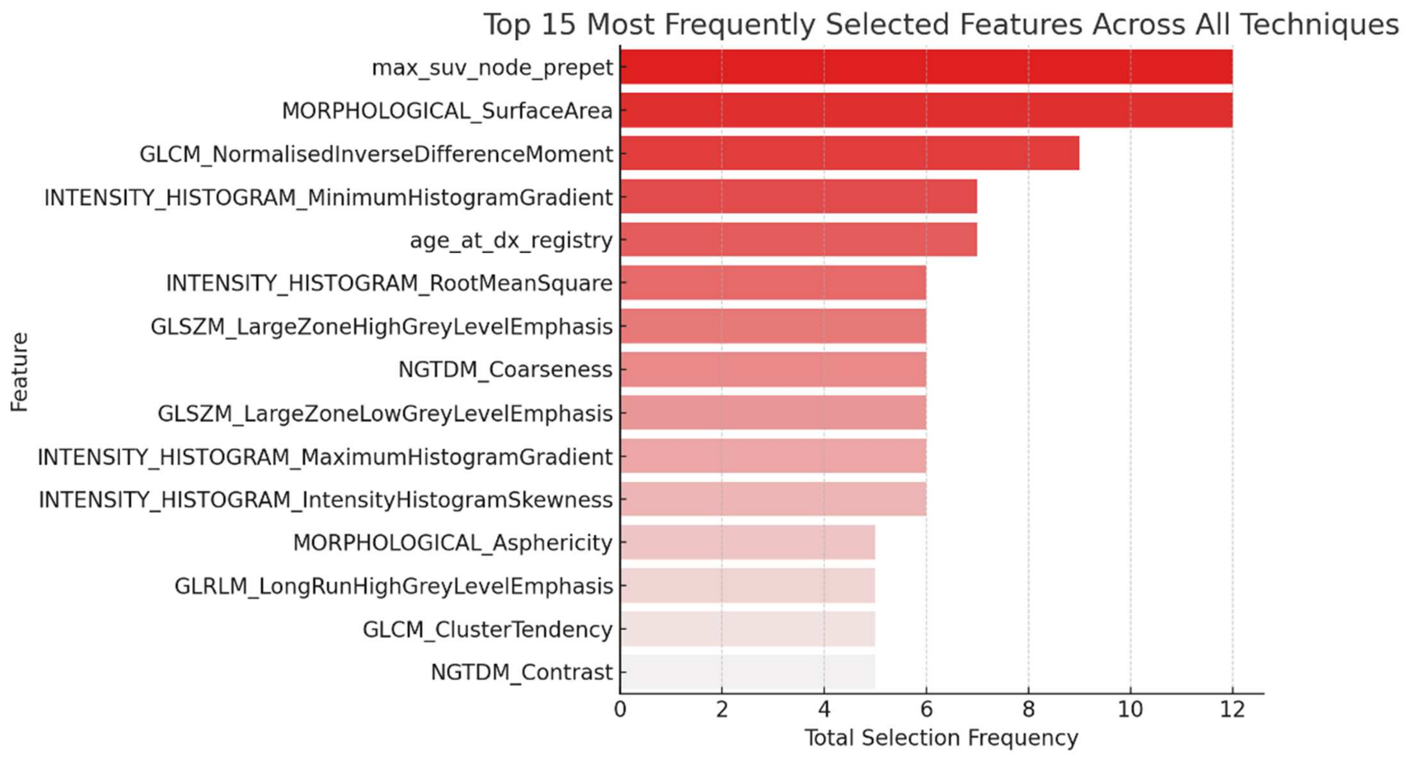

Figure S2. The most frequently selected features in this study (not on the external center)

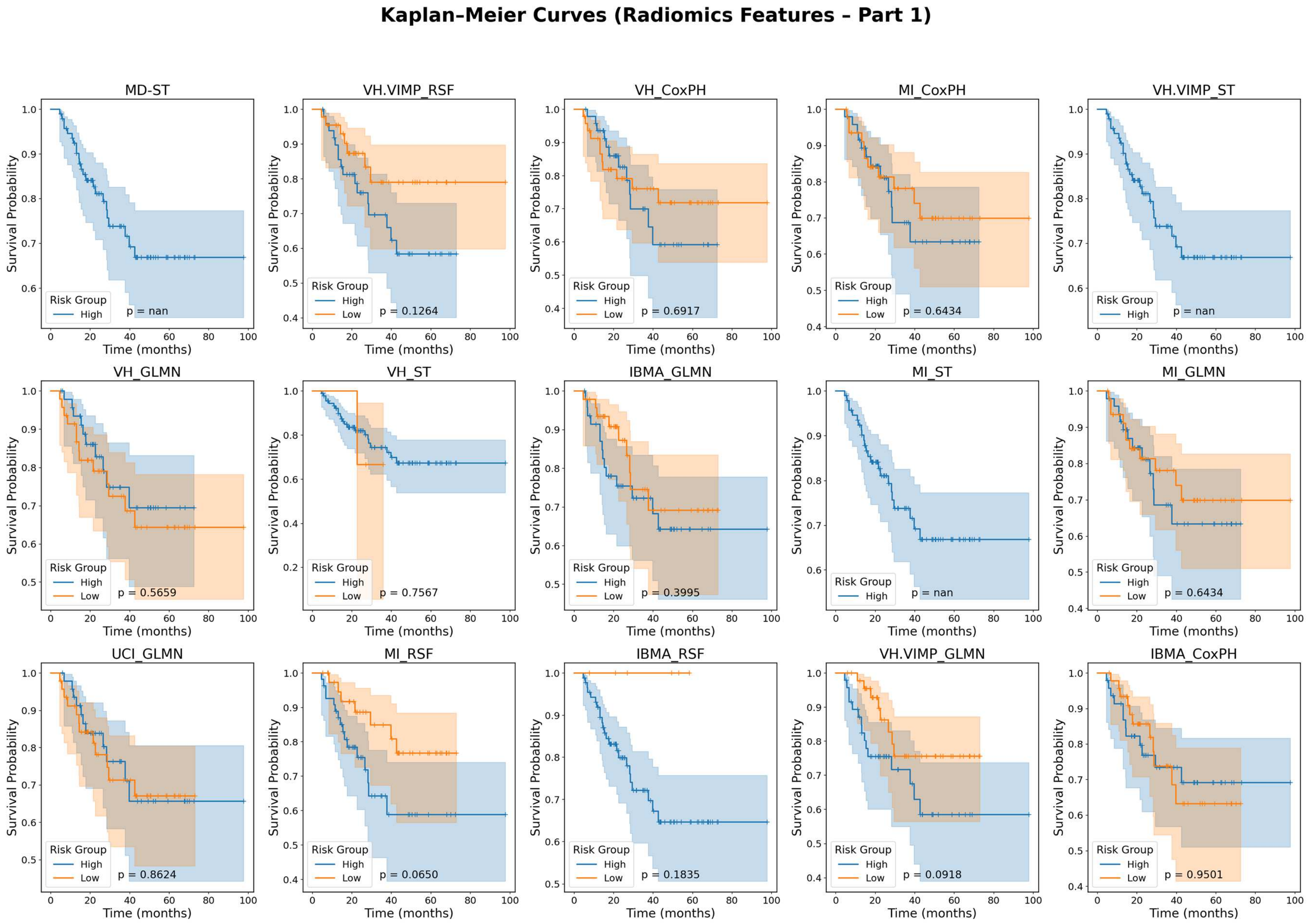
Kaplan–Meier Curves (Radiomics Features – Part 1)
MD-ST
p = nan
VH.VIMP_RSF
p = 0.1264
VH_CoxPH
p = 0.6917
MI_CoxPH
p = 0.6434
VH.VIMP_ST
p = nan
VH_GLMN
p = 0.5659
VH_ST
p = 0.7567
IBMA_GLMN
p = 0.3995
MI_ST
p = nan
MI_GLMN
p = 0.6434
UCI_GLMN
p = 0.8624
MI_RSF
p = 0.0650
IBMA_RSF
p = 0.1835
VH.VIMP_GLMN
p = 0.0918
IBMA_CoxPH
p = 0.9501
Risk Group
High
Low
Survival Probability
Time (months)

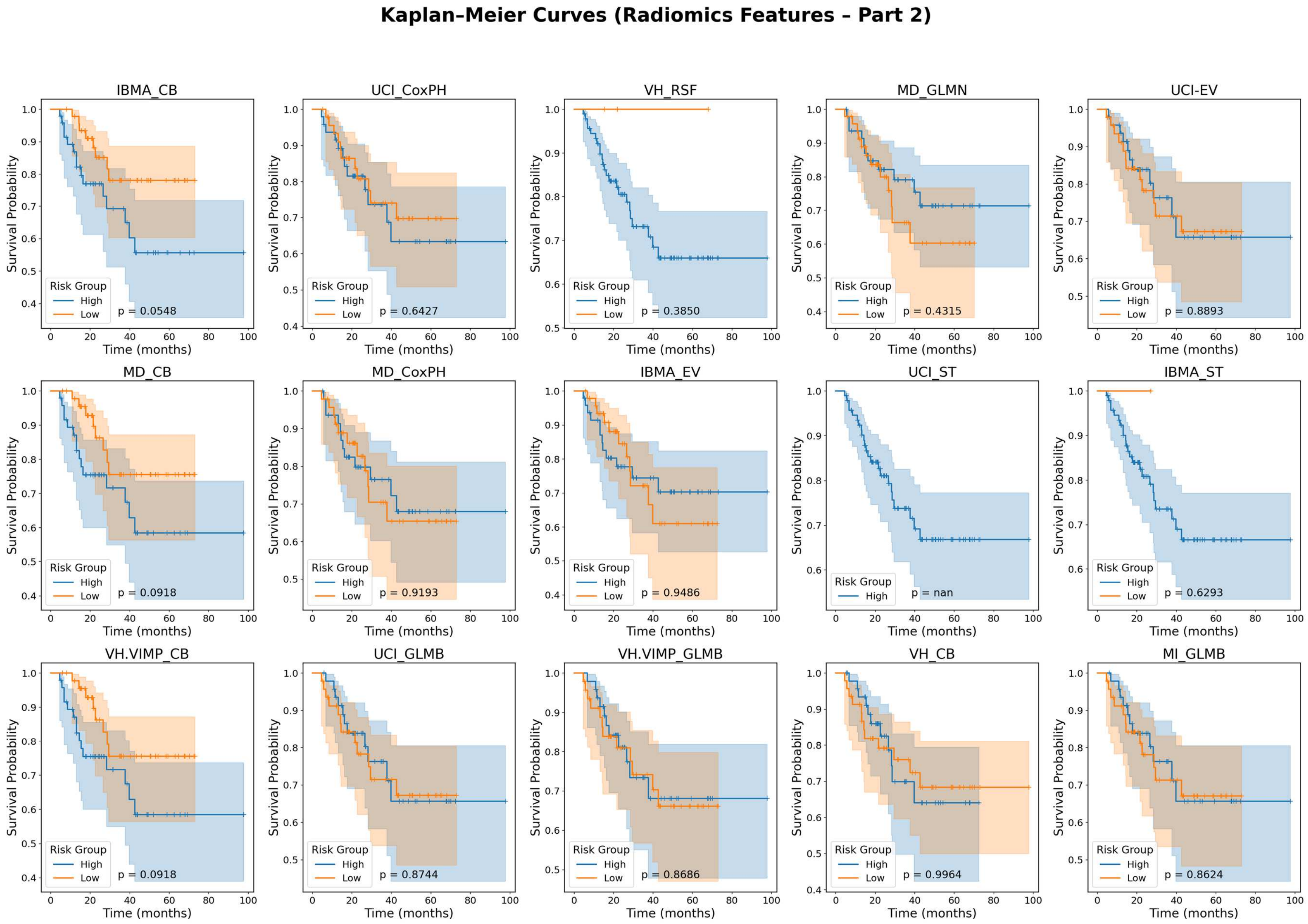

Kaplan-Meier Curves (Radiomics Features – Part 2)
IBMA_CB
p = 0.0548
UCI_CoxPH
p = 0.6427
VH_RSF
p = 0.3850
MD_GLMN
p = 0.4315
UCI-EV
p = 0.8893
MD_CB
p = 0.0918
MD_CoxPH
p = 0.9193
IBMA_EV
p = 0.9486
UCI_ST
p = nan
IBMA_ST
p = 0.6293
VH.VIMP_CB
p = 0.0918
UCI_GLMB
p = 0.8744
VH.VIMP_GLMB
p = 0.8686
VH_CB
p = 0.9964
MI_GLMB
p = 0.8624
Risk Group
High
Low
Survival Probability
Time (months)

Kaplan-Meier Curves (Radiomics Features – Part 3)

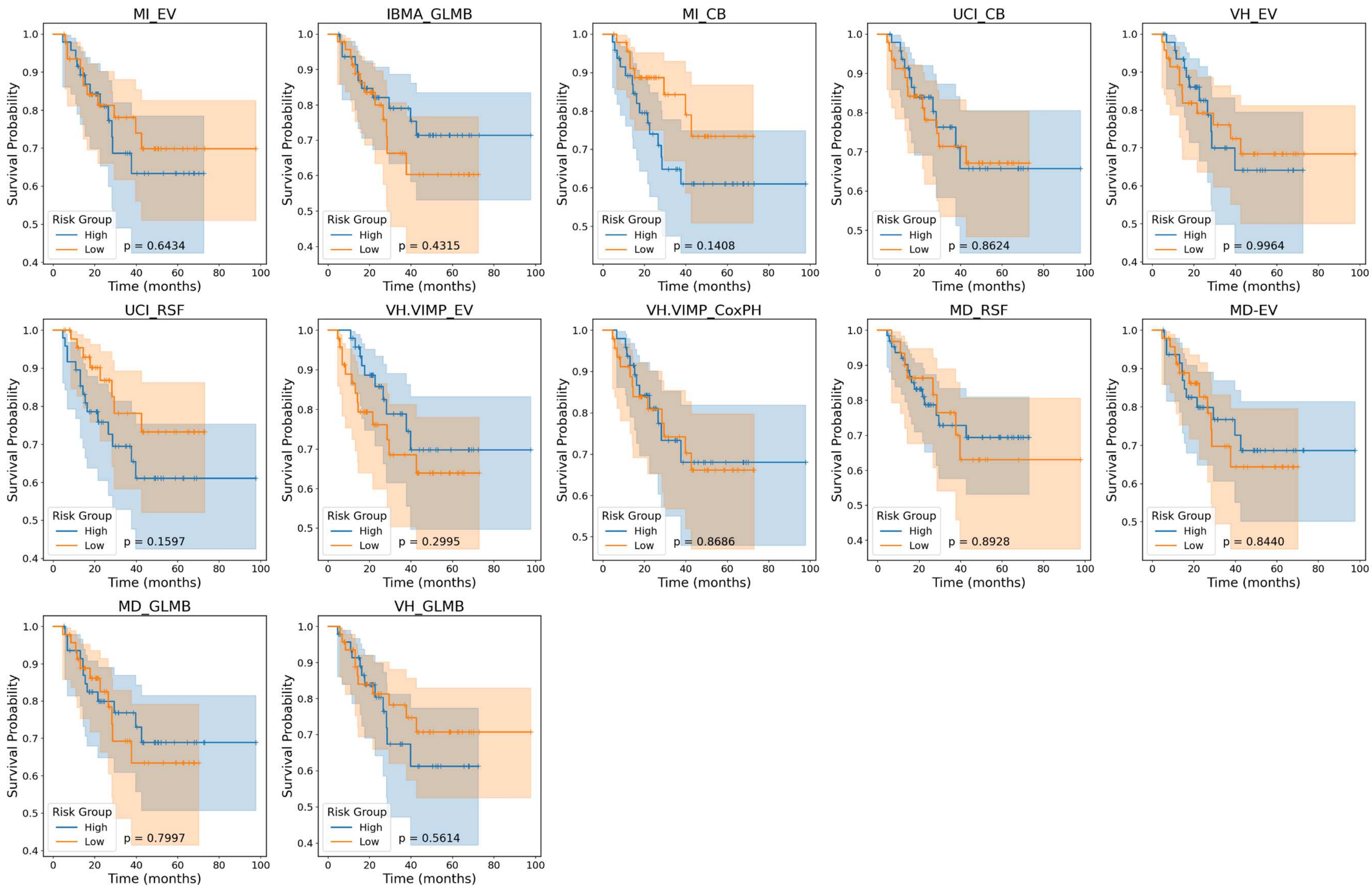

Figure S3. Kaplan–Meier curves corresponding to the combinations of feature selection and machine learning approaches with their corresponding log-rank p-values on data from the radiomics features from external center. Log-rank test p-values are reported for each comparison

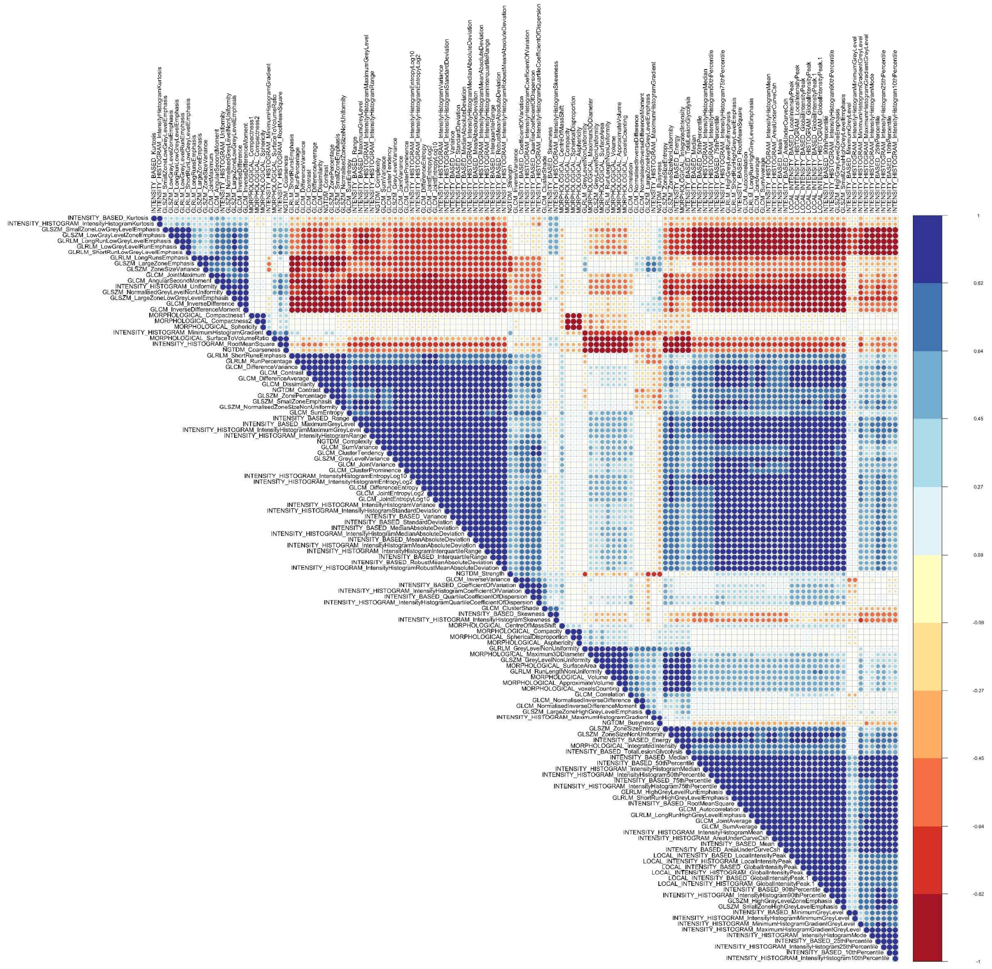

Figure S4. The correlation of the features
